# Estimation of the health economic benefit of widening pulmonary rehabilitation uptake and completion

**DOI:** 10.1177/14799731241307248

**Published:** 2024-12-08

**Authors:** Michael Steiner, James Mahon, Jonathan Fuld, Nick Hex

**Affiliations:** 1 Leicester NIHR Biomedical Research Centre – Respiratory, Department of Respiratory Sciences, 4488 University of Leicester, Leicester, UK; 2 357390 York Health Economics Consortium Ltd, University of York, Heslington, UK; 3 Victor Phillip Dahdaleh Heart and Lung Research Institute, 2152 University of Cambridge, Cambridge, UK

**Keywords:** Pulmonary rehabilitation, health economics, COPD, healthcare costs, willingness to pay

## Abstract

**Objectives:** Increasing uptake and completion of Pulmonary Rehabilitation in people with COPD has the potential to deliver health benefit and reduce health inequalities. We have quantified the cost-effectiveness of enhancing PR access and completion by reviewing the cost-effectiveness literature for PR in COPD. **Methods:** A literature review identified studies that provided cost-effectiveness evidence for PR compared to no PR. The key metrics of interest were healthcare resource use and cost savings, and quality adjusted life year (QALY) gains. Healthcare resource use data were valued using the UK NHS National Tariff 2022/23. From the literature search we identified the QALY gain resulting from completion of PR. The value of the QALY gain resulting from PR completion was calculated using the standard willingness-to-pay threshold of £20,000 considered by the UK National Institute for Health and care Excellence (NICE). **Results:** We estimated a QALY gain resulting from completion of PR of 0.065 and value of the QALY gain was therefore calculated to be £1300 per person completing PR. We estimated the 12 month reduction in hospitalisation following completion of PR to be 8.2% giving a total cost reduction per patient of £245. We therefore calculated that up to £1545 could be spent per person with COPD to deliver PR cost-effectively. **Conclusion:** Our analysis provides commissioners with the information they need to make informed decisions about planning and provision of PR. The data allows estimation of additional resources that could be deployed in addressing inequitable access to PR among disadvantaged and underserved populations whilst retaining cost effectiveness of the intervention.

## Introduction

Pulmonary Rehabilitation (PR) is of established effectiveness in the treatment of people with Chronic Obstructive Pulmonary Disease (COPD). The most recent Cochrane review recommended that further trials testing the effectiveness of the intervention are not needed and that research should focus on refining the content and availability of PR to maximise individual patient benefit.^1^ The American Thoracic Society (ATS) policy statement on Pulmonary Rehabilitation highlighted that despite these well documented benefits, many eligible patients are not referred, do not attend or do not complete treatment.^2^ For example, the UK national COPD audit estimated from primary care records that only approximately 15% of eligible patients were being referred for PR and sequences of national audits of PR services in the UK indicate that around one third of those referred for PR do not complete the programme.^3,4^ The UK NHS England 5-year vision for PR seeks to increase referral and uptake of PR married to a transformation of PR capacity to meet this demand.

There is evidence of health inequalities associated with access to PR for people with COPD.^5^ People with COPD in more socioeconomically deprived areas are less likely to complete PR than people in less deprived areas, but clinical outcomes are comparable for anyone completing their PR programme.^6,7^ Attendance at PR programmes in the UK is predominantly by people identifying as White-British.^4^ There is, therefore, a risk that in seeking to increase uptake of PR, there could also be a widening of health inequalities. The development of alternative models of PR delivery (for example digital or remote programmes) which has been accelerated by the COVID-19 pandemic might extend access to PR for patients who might struggle to travel to a PR centre but could also create inequity because of unequal access to digital technologies among patients.

If both the uptake and completion rates of PR could be increased in a cost-effective way for all people eligible (for example through targeted approaches to enhancing PR uptake in disadvantaged groups), there is potential to increase net population health benefits and reduce health inequalities. Indeed, it is likely that investment of resources in extending access to PR in currently underserved communities will be essential for efforts to increase net uptake of PR to succeed. For such investment to be justified, health commissioners need an understanding of the quantum of health gain that would arise from this commitment of resources.

This analysis was commissioned by the NHSE national respiratory programme to provide quantitative data informing the likely cost-effectiveness of delivering enhanced uptake and completion of PR. The analysis sought to estimate the resource envelope that commissioners could invest in delivering PR across the populations they serve that would be justifiable in terms of health benefit (cost/QALY). Specifically, we surveyed the relevant scientific literature to estimate;

1. The 12-month QALY gain arising from completion of PR.
2. The cost-savings to commissioners/payers over 12 months arising from the reduction in hospitalisation rates in patients completing PR (from UK tariff rates).
3. The net value of the QALY gain in relation to the standard willingness to pay threshold used by the UK National Institute for Health and Care Excellence (NICE)

The aim was to quantify the potential health economic benefits of enhancing uptake and completion of PR and thereby to provide commissioners with the information they need to make informed decisions about planning and provision of PR. Importantly, this information will allow estimates of the additional resources that could be deployed in addressing inequitable access to PR among disadvantaged and underserved populations whilst retaining the cost effectiveness of this investment.

## Methods

The starting point for the analysis was to review the work carried out by York Health Economics Consortium (YHEC) in 2011 on the cost-effectiveness of offering monetary incentives to primary care to increase the percentage of people with (COPD) being referred to a PR programme (report provided in online supplement).^8^ In its role as the National Collaborating Centre for Indicator Development to NICE, YHEC developed cost-effectiveness analyses for new indicators for the NHS Quality and Outcomes Framework (QOF). This work involved the development of net health benefit analysis of the impact of incentivising primary care to adopt NICE recommendations across a range of diseases and conditions. Net health benefit is a summary statistic that represents the impact on population health of introducing a new intervention. One of the areas examined by YHEC was the cost-effectiveness of offering monetary incentives to primary care to increase the percentage of people with COPD being referred to a PR programme. This report was based on evidence in two papers by Griffiths et al.^9,10^ In the original 2011 YHEC analysis for the QOF, the benefits of PR were estimated as reduced hospitalisations and improvement in QALYs based upon a health economic analysis of a randomised controlled trial of PR by Griffiths et al.^9^ For patients randomised to receive PR, Griffiths found a reduction in days of hospitalisation per patient over 12 months of 10.6 with a QALY gain of 0.03 per person. The benefits of PR estimated in Griffiths are based on an intent to treat (ITT) analysis and so are for patients who at least started PR and not only for those who completed PR.

Given that the QOF analysis was over 10 years old and based upon the NICE COPD Guideline from 2010, a pragmatic literature search was carried out to identify any cost-effectiveness evidence on PR that has been published subsequent to the 2011 analysis.

The search aimed to identify studies that provided cost-effectiveness evidence of PR compared to no PR. This was carried out using a targeted version of the filter developed by the University of York Centre for Reviews and Dissemination for identification of economic evaluations to include in NHS Economic Evaluation Database. The Medline database was searched. Broad disease and intervention identifiers were used to find evidence published since 2006, before the last COPD NICE recommendations were made that mentioned economic evaluations of pulmonary rehabilitation. Cost effectiveness studies in broad terms were considered eligible for selection and studies that compared different forms of PR were not considered relevant. The most relevant articles as defined by the database were screened by a single reviewer and the most appropriate studies were selected that matched the requirements of the proposed analysis.

The search terms and key words are provided in Table 1.

**Table 1.** Targeted MEDLINE search terms.

1	(pulmonary* or chronic obstructive pulmonary disorder or COPD) and rehabilitation
2	*Economics
3	Exp *“costs and cost analysis”
4	*Quality-adjusted life years
5	(Quality adjusted or adjusted life year*)
6	(qaly* or qald* or qale* or qtime*)
7	Limit to yr = “2006 -current”
8	Limit to english language

The key metrics of interest were healthcare resource use and cost savings, and quality adjusted life year (QALY) gains. The QALY is a summary outcome measure used to quantify the effectiveness of a particular intervention.^11^ Since the benefits of different interventions are multi-dimensional, QALYs have been designed to combine the impact of gains in quality of life and in quantity of life (i.e. life expectancy) associated with an intervention.

Any resource use data taken from the 2011 analysis or identified in any more recent relevant studies were valued using the National Tariff 2022/23 (https://www.england.nhs.uk/publication/national-tariff-payment-system-documents-annexes-and-supporting-documents/ (accessed 9 March 2023)).^12^ National Tariff values are an appropriate proxy for incremental costs that will be paid by Integrated Care Board (ICB) commissioning budgets. Tariff prices weighted by activity in the UK National Cost Collection suggests a price of £2854 per non-elective inpatient stay for COPD.^12^ Trim points when excess bed days can be applied are all 10 days or above and given that hospital stays for COPD are usually less than 10 days (The UK national COPD Audit reported a reduction from 9.8 days to 4.6 days). We therefore assumed that any reductions in lengths of stay resulting from PR completion would not result in significant reduction in excess bed day payments. Similarly, whilst the reduction in length of stay may trigger a lower payment under a reduced short stay emergency adjustment for some people, this will not be the case for the average person who is likely to stay in hospital for more than 2 days. To keep the analysis conservative the reduction in bed days was assumed to not reduce excess bed day payments by commissioners or to trigger a short stay emergency adjustment.

### Health economic impact assessment

The analysis parameters for the economic impact assessment were selected as they mirrored the parameters used in the previous analysis for the NICE QOF Committee.^8^ The 12-month gain to commissioners in terms of cost savings from PR was calculated from the estimated reduction in hospitalisation over 12 months multiplied by the average Tariff price per non-elective inpatient stay for COPD (£2854). The tariff value was considered to be a reasonable representative value for inpatient hospital stays relating to COPD.

Evidence from the literature was used to identify the QALY gain resulting from completion of PR. For simplicity and to account for the variable preservation of Quality of Life gains in the months following completion of PR, it was assumed that the utility gain would be maintained for 12 months but not beyond this timepoint, based on the evidence drawn from the literature. Mortality was not included in the estimation of QALY gains, to keep the analysis conservative and to account for uncertainties in the scientific evidence for the impact of PR on survival.

The value of the QALY gain resulting from PR completion was calculated using the standard willingness-to-pay threshold considered by the UK National Institute for Health and care Excellence (NICE). This is £20,000 per QALY, where willingness-to-pay is the valuation of health benefit in monetary terms.

The analysis was carried out in Microsoft Excel.

## Results

Summaries of the relevant literature^13–19^ identified in our search are provided in Table 2.

**Table 2.** Summary of the relevant literature identified subsequent to the YHEC 2011 report.

Author, year	Design	Comparators	QALY gain	Notes
Liu 2020^13^	Systematic review of cost effectiveness studies	10 studies included. Home, community or hospital-based PR vs usual care	Not given	PR conducted in different settings can potentially be cost-effective
Gillespie, 2013^14^	RCT in general practice in Ireland	Community based PR vs usual care	0.002	No reduction in healthcare resource use observed
Atsou, 2016^15^	Health economic (Markov) modelling	Modelled PR vs usual care	0.8 (lifetime)	Assumptions made that no impact on healthcare resource use and PR repeated every 2 years
Mosher 2022^16^	Health economic (Markov) modelling	Modelled PR after hospitalisation vs usual care	0.53 (lifetime)	Only post-hospitalisation setting considered
				Reduction in hospitalisation taken from Stefan, 2021 (8.6% absolute reduction)
				Assumed QoL gain Nolan 2016 (utility gain 0.065)
Nolan 2016^17^	EQ5D-5L utility index scores before and after PR	Cohort design (before and after)	0.065	Measured over 8 weeks of PR programme
Steiner 2017^18^	RCP national COPD audit series	Completers vs non-completers in PR cohort	N/A	Reduction of 13.9% hospitalisation rate 4.8 bed days in those completing PR
Stefan 2021^19^	Observational (retrospective) study of USA patients hospitalised with COPD	Cohort design (before and after)	N/A	8.2% reduction in hospitalisation over 12 months. 3.8-day reduction in length of hospital stay

### Summary of the literature identified

A systematic review by Liu et al (2021) was identified that compared the use of PR in different settings but included a number of cost-effectiveness studies of PR compared to usual care.^13^ The review identified two cost-effectiveness studies published post 2011 (after the YHEC analysis of PR for the QOF COPD indicator) that compared PR to usual care and where the authors considered that the results were robust enough to inform clinical practice.

Gillespie et al (2013) assessed the cost-effectiveness of a structured education PR programme in Ireland, via a trial based economic analysis of results from a randomised controlled trial with 22 weeks follow up from randomisation.^14^ Over the 22 weeks, PR was not found to reduce healthcare resource use but did improve quality of life with a QALY gain of 0.002.

Atsou et al (2016) constructed a lifetime economic model for patients receiving PR in France, with the assumption that PR increased quality of life but did not affect the exacerbation or mortality rate and, therefore, had no impact on hospitalisations or healthcare costs other than the cost of PR.^15^ A further assumption was that that PR would be required every 2 years to maintain effectiveness. A utility gain of 0.09 was estimated based upon a meta-analysis of PR trials that reported St George’s Respiratory Questionnaire (SGRQ) scores, which were then mapped to EQ5D scores. The total lifetime QALY gain estimated by the model with PR was 0.8 but this was reliant on PR being repeated every 2 years.

One cost-effectiveness study on PR was identified that was published after the Liu (2021) review. Mosher et al (2022) examined the lifetime cost-effectiveness of PR in US patients who had been hospitalised with COPD, but assumed the benefits of PR only occurred for 1 year.^16^ The reduction in 1-year hospitalisations (absolute reduction of 8.6%) and hospital bed days (3.8 days) with PR was taken from a propensity-score-matched analysis by Stefan et al (2021) of Medicare beneficiaries hospitalised with COPD who did and did not receive PR within 90 days of initial hospitalisation.

The lifetime QALY gains in Mosher et al (2022),^16^ estimated to be 0.53 QALYs for those completing PR within 90 days of admission, were based upon the reduction in mortality with PR in the first year and a utility response to PR of 0.065 from Nolan et al.^17^ In economic evaluation of healthcare interventions, utilities (also called health state preference values) are used to represent the strength of individuals’ preferences for different health states.^20^ When utility values are averaged over a population of responders they can be considered to be valuations of health states. Conventionally the valuations fall between 0 and 1, with 1 representing the valuation of a state of perfect health and 0 representing the valuation of death (non-existence).

The Nolan study was a quality of life study that reported EQ5D-5L utility index scores for 324 UK patients before and after completion of 8 weeks of PR.^17^ Mosher et al (2022) was the only study identified that sought to estimate the QALY gains over a lifetime due to the effectiveness of PR in reducing mortality.^16^

In 2017 the Royal College of Physicians (RCP) National COPD Audit undertook an analysis of audit data up to 2015 to examine the impact of completing PR on hospitalisation and hospital days.^18^ Patients who completed PR had a reduction of 13.9% in the rate of hospital admissions over 6 months and a length of stay 4.8 days shorter than those who did not complete PR.

Stefan et al (2021) estimated an absolute reduction in 12-month mortality of 6.7% with PR in their propensity-score-matched population.^19^ Mosher et al (2022) used that result (in patients who had been admitted to hospital) to estimate a total QALY gain of 0.53 QALYs, of which 0.065 was the direct QALY gain from PR.^16^ The absolute reduction in mortality for people who completed PR in the National COPD Audit was 2.7%, lower than the 6.7% reported by Stefan et al (2021). The potential QALY gain from mortality reduction with PR could be several times higher than the direct gain from PR.

### Health economic impact assessment

Data from Nolan et al.^17^ was used to estimate QALY gains rather than Mosher et al (2022), as it was a large UK-based study specifically looking at utility gains from PR rather than using a mapped utility gain approach. The estimated utility gain from Nolan from PR was 0.065 for patients who completed 8 weeks of PR. This results in a QALY gain from PR of 0.065. The standard willingness-to-pay threshold considered by NICE is £20,000 per QALY, where willingness-to-pay is the valuation of health benefit in monetary terms. This means that the value of the QALY gain can be considered to be £1300 per person completing PR (£20,000 × 0.065).

The analysis also used the reduction of 8.2% in hospitalisation over 12 months resulting from PR completion reported by Stefan et al.^19^ Although earlier Irish and French studies (Gillespie et al (2013) and Atsou et al (2016)) reported no change in healthcare resource use, more recent real-world evidence reported by Stefan et al (2021) using propensity-score matching does report reduced healthcare resources from completion of PR, backed up by the RCP COPD audit.^15^ The 12-month cost savings to commissioners from PR are, therefore, the assumed reduction in hospitalisation over 12 months (8.6%) multiplied by the average Tariff price per non-elective inpatient stay for COPD (£2854), giving a total cost reduction per patient of £245 (£2854 × 8.6%). By way of comparison, if absolute reductions in hospitalisation for people completing PR over 12 months matched those reported in the UK National COPD Audit over 6 months (13.9%), then the average reduction in spending on hospital care would increase to £397 (£2854 × 13.9%).

It was, therefore, calculated that for every person completing PR, up to £1545 could be spent per person with COPD eligible for PR, to deliver PR cost-effectively. This net benefit is achieved through an average cost reduction over 12 months of £245, and an average value of QALYs gained of £1300 (£1300 + £245).

## Discussion

In the current study, the cost effectiveness of completing PR for people with COPD has been quantified. Our analysis suggests an investment of up to £1545 per person with COPD would deliver value based on the NICE willingness-to-pay threshold. This net benefit figure arises from the combination of the cost/utility gain from completion of PR with the likely reduction in health resource utilisation arising from a reduction in 12-month hospitalisation rates. Given the unequivocal clinical benefits of completing PR, extending uptake and completion in people with COPD of this intervention is desirable. These data provide health commissioners and policy makers with information estimating the investment that would be justified in achieving this objective. We suggest this information can also be used to model the value of differential investment aimed at reducing inequality of uptake of PR in disadvantaged and/or underserved populations. For example, investment in reaching out to disadvantaged communities to offer PR services closer to their homes or providing translation services to facilitate access to PR for more diverse ethnic, religious and cultural groups might be a cost effective means to enhance uptake to PR. Such investment is likely to an effective means to enhance the population benefits of PR because evidence suggests that the outcome of PR in such populations is equivalent to less disadvantaged patients.

Our analysis suggests that PR is likely to result in a reduction in health resource utilisation, but that this might not be as great as has been reported in the Griffiths (2001) study.^9^ However, real world evidence (RWE) from the USA^19^ and the UK (from the RCP COPD Audit),^18^ suggests that PR can reduce both readmissions and length of stay. Whilst RWE can be prone to bias, the evidence from Stefan (2021) minimised bias through propensity-score-matching patients who did and did not have early PR^19^ This study also reported values for 12 months rather than 6 months which may give a better reflection of the efficacy of PR over time.

We used the Nolan et al (2016) study^17^ to estimate QALY gains (0.065 for patients who completed 8 weeks of PR), as it was a large UK-based study specifically looking at utility gains from PR, rather than the mapped utility gain used in Mosher.^16^ QALY gains from PR can also arise by reducing mortality and increasing life-years. However, given the uncertainty in the literature regarding the impact of PR on mortality and to maintain a conservative analysis, the base case for PR completion was based on QALY gains from utility gains rather than mortality gains.

The analysis has a number of limitations related to the assumptions made. A net benefit method was used, as opposed to alternatives such as budget impact analysis or return on investment, because the aim was to estimate the additional funding that could be justified to promote increased uptake of PR. A conservative approach was adopted to avoid over-complication and over-estimation of economic benefits of PR. Some of the benefits of PR were not included in the analysis (for example reductions in mortality and hospital length of stay) where evidence was less robust. Excluding these benefits in the assumptions does not imply they are not of potential importance. However, the approach has the advantage of providing a lower limit of justifiable spend on PR and it is possible that higher levels of investment might deliver health economic value. Whilst drawing on the published scientific literature internationally, the estimates of cost/utility gain employ calculations using UK payment tariffs and willingness-to-pay thresholds. The cost estimates may not be applicable in other countries with different health funding and payment arrangement. Indeed, willingness-to-pay thresholds in many other developed countries are deemed higher than the UK which would raise our estimates of cost-effective investment in those countries.^21^ Likewise, the patient populations and clinical settings studied in the identified literature varied; for example, some involved patients referred following discharge for acute exacerbations of COPD. Similarly, our estimates relate to the 12 month period following completion of PR. The uncertainty in extrapolating the impacts of PR across these populations and over longer durations is recognised. However, the objective of the analysis was not to generate a validated metric for cost/utility benefit (which would require prospective studies in specific populations) but to provide the parameters of investment that health services can use to inform commissioning decisions. We conducted a pragmatic literature search using an established search filter but recognise this was not a comprehensive systematic review which was beyond the scope and resources of the project. We recognise there might therefore be additional data available (for example unpublished studies) that might have influenced our estimates. Recognising the breadth of limitations to our analysis, we took a highly conservative approach to our estimates taking particular care not to overestimate the impact of reductions in hospitalisation or include potential reductions in mortality. This recognises the uncertainty in applying outcomes reported in studies such as Stefan et al conducted in the US to healthcare costs in the UK.

## Conclusions

We have quantified the value to the health system of PR completion for people with COPD. We suggest that investment by health commissioners in enhancing PR referral, uptake and completion, for example by targeting health inequalities in PR access, will provide significant and cost-effective population health benefit.

## Supplemental Material

**Supplemental Material -** Estimation of the health economic benefit of widening pulmonary rehabilitation uptake and completion Supplemental Material for Estimation of the health economic benefit of widening pulmonary rehabilitation uptake and completion by Michael Steiner, James Mahon, Jonathan Fuld and Nick Hex in Chronic Respiratory Disease.
