# Supplementary material for: Estimation of the health economic benefit of widening pulmonary rehabilitation uptake and completion
Source: Chron Respir Dis. 2024 Dec 8;21:14799731241307248. doi: 10.1177/14799731241307248 (PMC11626660; doi:10.1177/14799731241307248)
Supplement: Supplemental Material - Estimation of the health economic benefit of widening pulmonary rehabilitation uptake and completion [file sj-pdf-1-crd-10.1177_14799731241307248.pdf]

UNIVERSITY OF BIRMINGHAM AND UNIVERSITY OF YORK HEALTH ECONOMICS CONSORTIUM

(NICE EXTERNAL CONTRACTOR)

Health economic report on piloted indicator

QOF indicator area: COPD pulmonary rehabilitation

Potential output: Recommendations for NICE Menu

Contents

Introduction 2

Piloted indicator(s) 2

Economic rationale for the indicator 2

Objective 2

Type of health economic analysis 2

- Delivery cost of indicator 2
- Effectiveness of indicator 3
- Incremental cost-effectiveness ratio 3
- Eligible population 4
- Baseline level of achievement 4
- Population 4
- QOF Payments 5
- Societal value of a QALY 5
- QOF Points 5
- Thresholds 5

Results 5

Discussion 7

References 7

Appendix A: Net Benefit Analysis 8

Introduction

This briefing paper provides a summary of the economic evidence generated on the proposed pilot four COPD pulmonary rehabilitation indicator. The format of this paper is intended to provide the QOF Advisory Committee with sufficient information upon which to make a recommendation on whether the indicator is economically justifiable.

Piloted indicator

The percentage of patients with COPD and Medical Research Council (MRC) Dyspnoea Scale ≥3 at any time in the preceding 15 months, with a record of a referral to a pulmonary rehabilitation programme (excluding patients on the palliative care register).

Economic rationale for the indicator

The direct costs of COPD to the NHS are estimated to be close to £1 billion (1). There is evidence that pulmonary rehabilitation is a cost effective intervention for COPD, raising quality of life by reducing symptoms and potentially lowering hospitalisations for COPD exacerbation [1]

Objective

To evaluate whether the proposed indicator represents a cost effective use of NHS resources.

Type of health economic analysis

An indicative net benefit approach is applied with a one year time horizon.

Delivery cost of indicator

The cost of delivering the indicator needs to take into account the cost of discussing pulmonary rehabilitation with a patient and making the referral as well as the cost of the rehabilitation itself.

We have assumed that discussing rehabilitation and referring is undertaken through the equivalent of a GP consultation which lasts 17.2 minutes at a cost of £53, extracted from the Unit Costs of Health and Social Care 2010 [4].

The cost of rehabilitation itself varies in the literature. A study by Jones from 2002 [2] suggested costs were between £200 and £400 per patient and in a trial of primary care pulmonary rehabilitation they found costs to be £220 per completed patient which decreased the more patients were given the intervention. In a study by Griffiths [3] on hospital based rehabilitation, the cost was £712 per patient. As the Griffiths study forms the basis of much of our analysis we have taken this cost at baseline and increased it by inflation (HCHS) [4] to 2011 prices to give a cost of £1011. This is clearly at the upper end of potential costs in the literature and the incremental cost of rehabilitation was explored in sensitivity analysis.

In the analysis by Griffiths it was found that due to a reduction in hospitalisations and other healthcare use that pulmonary rehabilitation generated a saving over usual care of the order of £164 per patient. Most of this saving arose from a reduction in hospitalisations saving £804 per patient [3].

The NICE guidance states that whilst accepting the findings of the Griffiths study, there was conflicting evidence that pulmonary rehabilitation reduced hospitalisations. A second cost effectiveness study on pulmonary rehabilitation identified by NICE by the Wessex Institute could not be accessed in full form but NICE reported that they found a cost per QALY of £2000-£8000 per patient. NICE concluded that the trend in data is that pulmonary rehabilitation does reduce hospitalisations and that there is good evidence that it is cost effective against the willingness to pay threshold for a QALY.

For the purposes of the analysis we have taken assumed that there is a saving of £500 per patient from reduced hospitalisation but this is explored in sensitivity analysis over a range of £0-£1000.

Not all patients will survive until the end of treatment. However, mortality rates during rehabilitation could not be found and so for simplicity were assumed to be zero. QALYs in the model implicitly take into account mortality being taken from a trial where some patients died (although mortality was not explicit in the paper). This means that whilst benefits have been adjusted for mortality, costs have not skewing the costs in our analysis to be higher than they should be.

Not all patients offered rehabilitation will start rehabilitation even if referred. We have assumed at baseline that 80% of patients do accept treatment but this was explored in sensitivity analysis between 50% and 100% of patients.

The incremental annual cost of discussing pulmonary rehabilitation with patients with COPD and referring to pulmonary rehabilitation in comparison to usual care was estimated to be £461.80.

Effectiveness of indicator

As stated above, pulmonary rehabilitation is assumed to be effective at reducing hospitalisation and this is incorporated into how the model was presented.

NICE found that pulmonary rehabilitation has also been found to improve quality of life. The Griffiths study found that using the SF-36 questionnaire pulmonary rehabilitation generated 0.03 QALYs per patient. This gain in QALYs is used in our model, adjusted for our assumption that only 80% of patients at baseline accept a referral onto a rehabilitation programme. This means that at baseline the QALY gain per eligible patient is 0.024. This is varied in sensitivity analysis by +-50%.

The incremental QALY gain of pulmonary rehabilitation per eligible patient in comparison to usual care was estimated to be 0.024.

Incremental cost-effectiveness ratio

The NICE Guidance on COPD reported findings from the Griffiths paper that found pulmonary rehabilitation was both cost saving and improved quality of life. It also reported findings from the Wessex Institute of a cost per QALY between £2,000 and £8,000 per QALY.

Figure 1: Incremental cost-effectiveness ratio

$$ICER = \frac{Cost_{Treatment} - Cost_{Alternative}}{Effect_{Treatment} - Effect_{Alternative}}$$

Eligible population

The eligible population are patients with COPD with Dyspnoea Scale ≥3 and not receiving palliative care.

As the indicator refers to patients who had this diagnosis at any time in the preceding 15 months, the annual eligible population should be adjusted to 80% of the total population with COPD. However, in the first year of the indicator for a practice that had not been referring patients the effective population would be all patients with COPD and Dyspnoea Scale ≥3. We therefore have assumed that the eligible population is the number of patients on practice COPD registers with Dyspnoea Scale ≥3, but the size of the eligible population and the impact on cost effectiveness of the indicator is explored in sensitivity analysis.

The percentage of patients diagnosed with COPD is reported to be 1.5%, although it is acknowledged that this is probably an underestimate of true prevalence of the condition [1].

Whilst the prevalence of diagnosed COPD can be established, data on the proportion of patients with Dyspnoea Scale ≥3 or who are receiving palliative care could not be found. As a baseline we assumed that 50% of patients would not be eligible for pulmonary rehabilitation and we explored this with sensitivity analysis for a range from 25% to 75%.

Using the above assumptions, at baseline the eligible population was assumed to be 0.75%.

Baseline level of achievement

Data from the pilot sites suggested that this was largely new work, with the indicator being achieved for 9.98% of eligible patients at the beginning of the pilot, rising to 18.25% at its conclusion. We have assumed that baseline achievement is 25%.

Population

In the base case, the threshold analysis of the proposed indicator was conducted based on the total practice population registered with practices in England, that is, 8,228 practices with a mean practice size of 6,297 [5].

Table 1: Practice information for all UK members

Country	Number of practices	Number of patients
England	8,228	6,297
Scotland	1,014	5,122
Wales	488	6,146
Northern Ireland	357	5,011

QOF Payments

Each QOF point is assumed to result in a payment of £133.76. This is the forecast value per point in England during 2011/12 (source; Information Centre).

Table 2: Value per point for all UK members (most recently available)

Country	Value per point
England	£133.76
Scotland	£130.46
Wales	£133.72
Northern Ireland	£125.04

Societal value of a QALY

The expected increase in quality adjusted life year (QALY) will be costed at both £20,000 and £25,000 per QALY. This is based on the bottom and the middle of the range £20,000 - £30,000, below which NICE generally considers something to be cost effective.

QOF Points

The economic analysis considers the cost-effectiveness of incentivising the proposed activity over a range of QOF points. The range of QOF points evaluated was agreed by NICE, YHEC and the economic sub-group to justify the practice successfully completing the activity.

In the base case analysis, 5 points were allocated to the proposed indicator. Sensitivity analysis will be followed out between the agreed lower and upper bounds of 2 and 10 points (i.e. the range evaluated).

Thresholds

The minimum threshold is set to 40% and the incentivised payments increase linearly up to the maximum threshold of 90%.

Results (assuming a value per QALY of £25,000)

The indicative net benefit analysis suggests that the indicator is highly cost effective, with QOF payments up to the upper bound of 10 points warranted on economic grounds (Appendix A). The increase in quality of life offered by advice and treatment

outweighs the additional healthcare costs in a net benefit analysis if the value per QALY is assumed to be £25,000.

Sensitivity analysis shows the findings are sensitive to a removal of any cost savings obtained from pulmonary rehabilitation which reduces secondary healthcare usage such as hospitalisations. (Appendix B). The indicator ceases to be justifiable on economic grounds at 5 points and 90% achievement when the incremental cost of delivering the indicator reaches £578 per patient.

The findings are also sensitive to a 50% reduction in the assumed utility gains (Appendix C) at which point again the indicator cannot be justified on economic grounds. The indicator ceases to be justifiable on economic grounds at 5 points and 90% achievement when the utility gain per patient falls to 0.019.

Sensitivity analysis explored how conclusions changed if the eligible population fell because only 25% of COPD patients were eligible for pulmonary rehabilitation (Appendix D). This made no difference to the overall findings. The eligible population would have to fall to 1.18% before the indicator could not be recommended on economic grounds at a baseline of 5 points.

If the assumptions underpinning this analysis hold, then due to the potential size of the eligible population and the relatively low cost of the intervention (after cost savings from reduced hospitalisation) when compared to potential quality of life gains, there is a strong economic case for the indicator at a baseline of 5 points and 90% achievement. There are economic grounds to award up to the maximum QOF points appropriate for this indicator, i.e. 10 points.

Results (assuming a value per QALY of £20,000)

Assuming a value per QALY of £20,000, the indicative net benefit analysis suggests that the indicator is not cost effective at five points and 90% achievement, with QOF payments only up to four points warranted on economic grounds (Appendix E). At five points, the indicator becomes cost effective when the value of a QALY rises to £20,158.

Sensitivity analysis shows the findings are sensitive to a removal of any cost savings obtained from pulmonary rehabilitation which reduces secondary healthcare usage such as hospitalisations. At that point the indicator is not justifiable at any number of points. (Appendix F). The indicator becomes justifiable on economic grounds at 5 points and 90% achievement when the incremental net cost of delivering the indicator falls by just £4 to £458 per patient.

The findings are also sensitive to a 50% reduction in the assumed utility gains (Appendix G) at which point the indicator is not justifiable at any number of points. The indicator becomes justifiable on economic grounds at 5 points and 90% achievement when the utility gain per patient rises by less than 0.001 of a QALY.

Sensitivity analysis explored how conclusions changed if the eligible population fell because only 25% of COPD patients were eligible for pulmonary rehabilitation (Appendix H). This reduction results in the indicator not being justifiable on economic grounds at 5 points although still justifiable at 2 points.

Discussion

In the NICE guidelines the conclusion was reached that the available evidence was that pulmonary rehabilitation was cost effective for patients with COPD. Our conclusions support this, but at baseline the findings are dependent on pulmonary rehabilitation reducing secondary healthcare resource usage, notably hospitalisations. The literature is not unambiguously supportive of this. However, it should also be pointed out that our estimate of the cost of pulmonary rehabilitation may be overly pessimistic as it is at the upper end of reported costs for the intervention.

It should also be noted that the Griffiths paper was also in relation to people with lung disease, although patients were predominantly those with COPD. It is not thought that this would affect findings and NICE was critical of the study on that basis, in their guidance on COPD.

With a QALY value of £25,000 and ignoring any potential cost savings from pulmonary rehabilitation then under our baseline assumptions the indicator is justifiable on economic grounds at 5 points.

With a QALY value of £20,000, pulmonary rehabilitation is not economically justifiable on economic grounds at 5 points and 90% achievement. However, the case is borderline with only very small decreases in costs or increases in QALYs required for the indicator to be economically justified at that level.

On balance the modelling points to the indicator being economically justified. However, this depends on whether the scale of the utility gain and assumed cost savings at baseline are considered to be cautious rather than over optimistic.

References

[1] National Clinical Guideline Centre. (2010) Chronic obstructive pulmonary disease: management of chronic obstructive pulmonary disease in adults in primary and secondary care. London: National Clinical Guideline Centre

[2] A pilot study of pulmonary rehabilitation in primary care. Jones RCM, Copper S, Riley O and Dobbs F. British Journal of General Practice 2002.

[3] Cost effectiveness of an outpatient multidisciplinary pulmonary rehabilitation programme. Griffiths TL, Phillips CJ, Davies S, Burr ML, Campbell IA. Thorax 2001

[4] Unit Costs of Health & Social Care 2010. Personal Social Services Research Unit (PSSRU). Complied by Lesley Curtis. University of Kent.

[5] General Practice Trends in the UK. NHS Information Centre. Published 22 March 2011.

Appendix A: Net Benefit Base Case Analysis

Pilot 4 - Referral to Pulmonary Rehab for COPD: Net Benefit Analysis

Value per point achieved	£133.76	Societal value of a QALY	£25,000		
Number of practices	8,228				
Mean practice population	6,297				
		**Basline achievement**			
Minimum threshold	40%	Eligible population (mean % of practice population)	0.750%		£462
Maximum threshold	90%	Baseline achievement (mean % of eligible patients)	25.0%		0.024

**Points**	2	3	4	5	6	7	8	9	10

National totals

Expected Achievement	QOF payments (£000s)									Change in treatment cost (£)	Change in QALYs
30%	£0	£0	£0	£0	£0	£0	£0	£0	£0	£8,972,494	466
35%	£0	£0	£0	£0	£0	£0	£0	£0	£0	£17,944,988	933
40%	£0	£0	£0	£0	£0	£0	£0	£0	£0	£26,917,482	1399
45%	£220	£330	£440	£550	£660	£770	£880	£991	£1,101	£35,889,976	1865
50%	£440	£660	£880	£1,101	£1,321	£1,541	£1,761	£1,981	£2,201	£44,862,470	2332
55%	£660	£991	£1,321	£1,651	£1,981	£2,311	£2,641	£2,972	£3,302	£53,834,964	2798
60%	£880	£1,321	£1,761	£2,201	£2,641	£3,082	£3,522	£3,962	£4,402	£62,807,457	3264
65%	£1,101	£1,651	£2,201	£2,751	£3,302	£3,852	£4,402	£4,953	£5,503	£71,779,951	3730
70%	£1,321	£1,981	£2,641	£3,302	£3,962	£4,622	£5,283	£5,943	£6,603	£80,752,445	4197
75%	£1,541	£2,311	£3,082	£3,852	£4,622	£5,393	£6,163	£6,934	£7,704	£89,724,939	4663
80%	£1,761	£2,641	£3,522	£4,402	£5,283	£6,163	£7,044	£7,924	£8,805	£98,697,433	5129
85%	£1,981	£2,972	£3,962	£4,953	£5,943	£6,934	£7,924	£8,915	£9,905	£107,669,927	5596
90%	£2,201	£3,302	£4,402	£5,503	£6,603	£7,704	£8,805	£9,905	£11,006	£116,642,421	6062
95%	£2,201	£3,302	£4,402	£5,503	£6,603	£7,704	£8,805	£9,905	£11,006	£125,614,915	6528
100%	£2,201	£3,302	£4,402	£5,503	£6,603	£7,704	£8,805	£9,905	£11,006	£134,587,409	6995

Net Benefit (£000s)

30%	£2,685	£2,685	£2,685	£2,685	£2,685	£2,685	£2,685	£2,685	£2,685
35%	£5,370	£5,370	£5,370	£5,370	£5,370	£5,370	£5,370	£5,370	£5,370
40%	£8,055	£8,055	£8,055	£8,055	£8,055	£8,055	£8,055	£8,055	£8,055
45%	£10,520	£10,410	£10,300	£10,190	£10,080	£9,970	£9,860	£9,750	£9,640
50%	£12,985	£12,765	£12,545	£12,325	£12,105	£11,885	£11,665	£11,445	£11,225
55%	£15,451	£15,120	£14,790	£14,460	£14,130	£13,800	£13,469	£13,139	£12,809
60%	£17,916	£17,475	£17,035	£16,595	£16,155	£15,714	£15,274	£14,834	£14,394
65%	£20,381	£19,830	£19,280	£18,730	£18,179	£17,629	£17,079	£16,529	£15,978
70%	£22,846	£22,185	£21,525	£20,865	£20,204	£19,544	£18,884	£18,223	£17,563
75%	£25,311	£24,540	£23,770	£22,999	£22,229	£21,459	£20,688	£19,918	£19,147
80%	£27,776	£26,895	£26,015	£25,134	£24,254	£23,373	£22,493	£21,612	£20,732
85%	£30,241	£29,250	£28,260	£27,269	£26,279	£25,288	£24,298	£23,307	£22,317
90%	£32,706	£31,605	£30,505	£29,404	£28,303	£27,203	£26,102	£25,002	£23,901
95%	£35,391	£34,290	£33,190	£32,089	£30,989	£29,888	£28,787	£27,687	£26,586
100%	£38,076	£36,975	£35,875	£34,774	£33,674	£32,573	£31,473	£30,372	£29,271

Where the net benefit produces a non-negative outcome then it is cost effective for the NHS to adopt the indicator.

When this is the case, the cells are highlighted with a yellow background.

Appendix B: Net Benefit Analysis Assuming No Reduction in Secondary Healthcare Costs

Pilot 4 - Referral to Pulmonary Rehab for COPD: Net Benefit Analysis

Value per point achieved	£133.76	Societal value of a QALY	£25,000
Number of practices	8,228		
Mean practice population	6,297		

Basline achievement

Minimum threshold	40%	Eligible population (mean % of practice population)	0.750%	£954
Maximum threshold	90%	Baseline achievement (mean % of eligible patients)	25.0%	0.024

National totals

**Expected Achievement**	**QOF payments (£000s)**									**Change in treatment cost (£)**	**Change in QALYs**
**Points**	2	3	4	5	6	7	8	9	10		
30%	£0	£0	£0	£0	£0	£0	£0	£0	£0	£18,535,641	466
35%	£0	£0	£0	£0	£0	£0	£0	£0	£0	£37,071,283	933
40%	£0	£0	£0	£0	£0	£0	£0	£0	£0	£55,606,924	1399
45%	£220	£330	£440	£550	£660	£770	£880	£991	£1,101	£74,142,566	1865
50%	£440	£660	£880	£1,101	£1,321	£1,541	£1,761	£1,981	£2,201	£92,678,207	2332
55%	£660	£991	£1,321	£1,651	£1,981	£2,311	£2,641	£2,972	£3,302	£111,213,848	2798
60%	£880	£1,321	£1,761	£2,201	£2,641	£3,082	£3,522	£3,962	£4,402	£129,749,490	3264
65%	£1,101	£1,651	£2,201	£2,751	£3,302	£3,852	£4,402	£4,953	£5,503	£148,285,131	3730
70%	£1,321	£1,981	£2,641	£3,302	£3,962	£4,622	£5,283	£5,943	£6,603	£166,820,773	4197
75%	£1,541	£2,311	£3,082	£3,852	£4,622	£5,393	£6,163	£6,934	£7,704	£185,356,414	4663
80%	£1,761	£2,641	£3,522	£4,402	£5,283	£6,163	£7,044	£7,924	£8,805	£203,892,055	5129
85%	£1,981	£2,972	£3,962	£4,953	£5,943	£6,934	£7,924	£8,915	£9,905	£222,427,697	5596
90%	£2,201	£3,302	£4,402	£5,503	£6,603	£7,704	£8,805	£9,905	£11,006	£240,963,338	6062
95%	£2,201	£3,302	£4,402	£5,503	£6,603	£7,704	£8,805	£9,905	£11,006	£259,498,980	6528
100%	£2,201	£3,302	£4,402	£5,503	£6,603	£7,704	£8,805	£9,905	£11,006	£278,034,621	6995

Net Benefit (£000s)

30%	-£6,878	-£6,878	-£6,878	-£6,878	-£6,878	-£6,878	-£6,878	-£6,878	-£6,878
35%	-£13,756	-£13,756	-£13,756	-£13,756	-£13,756	-£13,756	-£13,756	-£13,756	-£13,756
40%	-£20,634	-£20,634	-£20,634	-£20,634	-£20,634	-£20,634	-£20,634	-£20,634	-£20,634
45%	-£27,732	-£27,842	-£27,952	-£28,062	-£28,172	-£28,282	-£28,392	-£28,503	-£28,613
50%	-£34,830	-£35,050	-£35,270	-£35,491	-£35,711	-£35,931	-£36,151	-£36,371	-£36,591
55%	-£41,928	-£42,259	-£42,589	-£42,919	-£43,249	-£43,579	-£43,909	-£44,240	-£44,570
60%	-£49,026	-£49,467	-£49,907	-£50,347	-£50,787	-£51,228	-£51,668	-£52,108	-£52,548
65%	-£56,125	-£56,675	-£57,225	-£57,775	-£58,326	-£58,876	-£59,426	-£59,977	-£60,527
70%	-£63,223	-£63,883	-£64,543	-£65,204	-£65,864	-£66,524	-£67,185	-£67,845	-£68,506
75%	-£70,321	-£71,091	-£71,862	-£72,632	-£73,402	-£74,173	-£74,943	-£75,714	-£76,484
80%	-£77,419	-£78,299	-£79,180	-£80,060	-£80,941	-£81,821	-£82,702	-£83,582	-£84,463
85%	-£84,517	-£85,508	-£86,498	-£87,489	-£88,479	-£89,470	-£90,460	-£91,451	-£92,441
90%	-£91,615	-£92,716	-£93,816	-£94,917	-£96,018	-£97,118	-£98,219	-£99,319	-£100,420
95%	-£98,493	-£99,594	-£100,694	-£101,795	-£102,896	-£103,996	-£105,097	-£106,197	-£107,298
100%	-£105,371	-£106,472	-£107,572	-£108,673	-£109,774	-£110,874	-£111,975	-£113,075	-£114,176

Where the net benefit produces a non-negative outcome then it is cost effective for the NHS to adopt the indicator.

When this is the case, the cells are highlighted with a yellow background.

Appendix C: Net Benefit Analysis Assuming 50% Reduction in Utility

Pilot 4 - Referral to Pulmonary Rehab for COPD: Net Benefit Analysis

Value per point achieved	£133.76	Societal value of a QALY	£25,000		
Number of practices	8,228				
Mean practice population	6,297				
		**Basline achievement**			
Minimum threshold	40%	Eligible population (mean % of practice population)	0.750%		£462
Maximum threshold	90%	Baseline achievement (mean % of eligible patients)	25.0%		0.012

National totals

Expected Achievement	QOF payments (£000s)									Change in treatment cost (£)	Change in QALYs
**Points**	2	3	4	5	6	7	8	9	10		
30%	£0	£0	£0	£0	£0	£0	£0	£0	£0	£8,976,380	233
35%	£0	£0	£0	£0	£0	£0	£0	£0	£0	£17,952,760	466
40%	£0	£0	£0	£0	£0	£0	£0	£0	£0	£26,929,139	699
45%	£220	£330	£440	£550	£660	£770	£880	£991	£1,101	£35,905,519	933
50%	£440	£660	£880	£1,101	£1,321	£1,541	£1,761	£1,981	£2,201	£44,881,899	1166
55%	£660	£991	£1,321	£1,651	£1,981	£2,311	£2,641	£2,972	£3,302	£53,858,279	1399
60%	£880	£1,321	£1,761	£2,201	£2,641	£3,082	£3,522	£3,962	£4,402	£62,834,659	1632
65%	£1,101	£1,651	£2,201	£2,751	£3,302	£3,852	£4,402	£4,953	£5,503	£71,811,038	1865
70%	£1,321	£1,981	£2,641	£3,302	£3,962	£4,622	£5,283	£5,943	£6,603	£80,787,418	2098
75%	£1,541	£2,311	£3,082	£3,852	£4,622	£5,393	£6,163	£6,934	£7,704	£89,763,798	2332
80%	£1,761	£2,641	£3,522	£4,402	£5,283	£6,163	£7,044	£7,924	£8,805	£98,740,178	2565
85%	£1,981	£2,972	£3,962	£4,953	£5,943	£6,934	£7,924	£8,915	£9,905	£107,716,558	2798
90%	£2,201	£3,302	£4,402	£5,503	£6,603	£7,704	£8,805	£9,905	£11,006	£116,692,937	3031
95%	£2,201	£3,302	£4,402	£5,503	£6,603	£7,704	£8,805	£9,905	£11,006	£125,669,317	3264
100%	£2,201	£3,302	£4,402	£5,503	£6,603	£7,704	£8,805	£9,905	£11,006	£134,645,697	3497

Net Benefit (£000s)

30%	-£3,148	-£3,148	-£3,148	-£3,148	-£3,148	-£3,148	-£3,148	-£3,148	-£3,148
35%	-£6,295	-£6,295	-£6,295	-£6,295	-£6,295	-£6,295	-£6,295	-£6,295	-£6,295
40%	-£9,443	-£9,443	-£9,443	-£9,443	-£9,443	-£9,443	-£9,443	-£9,443	-£9,443
45%	-£12,810	-£12,920	-£13,030	-£13,141	-£13,251	-£13,361	-£13,471	-£13,581	-£13,691
50%	-£16,178	-£16,398	-£16,618	-£16,838	-£17,059	-£17,279	-£17,499	-£17,719	-£17,939
55%	-£19,546	-£19,876	-£20,206	-£20,536	-£20,866	-£21,197	-£21,527	-£21,857	-£22,187
60%	-£22,913	-£23,354	-£23,794	-£24,234	-£24,674	-£25,115	-£25,555	-£25,995	-£26,435
65%	-£26,281	-£26,831	-£27,382	-£27,932	-£28,482	-£29,033	-£29,583	-£30,133	-£30,683
70%	-£29,649	-£30,309	-£30,969	-£31,630	-£32,290	-£32,950	-£33,611	-£34,271	-£34,932
75%	-£33,016	-£33,787	-£34,557	-£35,328	-£36,098	-£36,868	-£37,639	-£38,409	-£39,180
80%	-£36,384	-£37,265	-£38,145	-£39,025	-£39,906	-£40,786	-£41,667	-£42,547	-£43,428
85%	-£39,752	-£40,742	-£41,733	-£42,723	-£43,714	-£44,704	-£45,695	-£46,685	-£47,676
90%	-£43,119	-£44,220	-£45,321	-£46,421	-£47,522	-£48,622	-£49,723	-£50,823	-£51,924
95%	-£46,267	-£47,368	-£48,468	-£49,569	-£50,669	-£51,770	-£52,870	-£53,971	-£55,072
100%	-£49,415	-£50,515	-£51,616	-£52,716	-£53,817	-£54,917	-£56,018	-£57,119	-£58,219

Where the net benefit produces a non-negative outcome then it is <u>cost effective</u> for the NHS to adopt the indicator.

When this is the case, the cells are highlighted with a yellow background.

Appendix D: Net Benefit Analysis Assuming Lower Estimate for Eligible Population

Pilot 4 - Referral to Pulmonary Rehab for COPD: Net Benefit Analysis

Value per point achieved	£133.76	Societal value of a QALY	£25,000
Number of practices	8,228		
Mean practice population	6,297		

Basline achievement

Minimum threshold	40%	Eligible population (mean % of practice population)	0.375%	£462
Maximum threshold	90%	Baseline achievement (mean % of eligible patients)	25.0%	0.024

National totals

Expected Achievement	QOF payments (£000s)									Change in treatment cost (£)	Change in QALYs
**Points**	2	3	4	5	6	7	8	9	10		
30%	£0	£0	£0	£0	£0	£0	£0	£0	£0	£4,488,190	233
35%	£0	£0	£0	£0	£0	£0	£0	£0	£0	£8,976,380	466
40%	£0	£0	£0	£0	£0	£0	£0	£0	£0	£13,464,570	699
45%	£220	£330	£440	£550	£660	£770	£880	£991	£1,101	£17,952,760	933
50%	£440	£660	£880	£1,101	£1,321	£1,541	£1,761	£1,981	£2,201	£22,440,949	1166
55%	£660	£991	£1,321	£1,651	£1,981	£2,311	£2,641	£2,972	£3,302	£26,929,139	1399
60%	£880	£1,321	£1,761	£2,201	£2,641	£3,082	£3,522	£3,962	£4,402	£31,417,329	1632
65%	£1,101	£1,651	£2,201	£2,751	£3,302	£3,852	£4,402	£4,953	£5,503	£35,905,519	1865
70%	£1,321	£1,981	£2,641	£3,302	£3,962	£4,622	£5,283	£5,943	£6,603	£40,393,709	2098
75%	£1,541	£2,311	£3,082	£3,852	£4,622	£5,393	£6,163	£6,934	£7,704	£44,881,899	2332
80%	£1,761	£2,641	£3,522	£4,402	£5,283	£6,163	£7,044	£7,924	£8,805	£49,370,089	2565
85%	£1,981	£2,972	£3,962	£4,953	£5,943	£6,934	£7,924	£8,915	£9,905	£53,858,279	2798
90%	£2,201	£3,302	£4,402	£5,503	£6,603	£7,704	£8,805	£9,905	£11,006	£58,346,469	3031
95%	£2,201	£3,302	£4,402	£5,503	£6,603	£7,704	£8,805	£9,905	£11,006	£62,834,659	3264
100%	£2,201	£3,302	£4,402	£5,503	£6,603	£7,704	£8,805	£9,905	£11,006	£67,322,848	3497

Net Benefit (£000s)

30%	£1,341	£1,341	£1,341	£1,341	£1,341	£1,341	£1,341	£1,341	£1,341
35%	£2,681	£2,681	£2,681	£2,681	£2,681	£2,681	£2,681	£2,681	£2,681
40%	£4,022	£4,022	£4,022	£4,022	£4,022	£4,022	£4,022	£4,022	£4,022
45%	£5,142	£5,032	£4,922	£4,812	£4,702	£4,592	£4,482	£4,372	£4,262
50%	£6,263	£6,043	£5,823	£5,603	£5,382	£5,162	£4,942	£4,722	£4,502
55%	£7,383	£7,053	£6,723	£6,393	£6,063	£5,733	£5,402	£5,072	£4,742
60%	£8,504	£8,064	£7,623	£7,183	£6,743	£6,303	£5,863	£5,422	£4,982
65%	£9,624	£9,074	£8,524	£7,974	£7,423	£6,873	£6,323	£5,772	£5,222
70%	£10,745	£10,085	£9,424	£8,764	£8,104	£7,443	£6,783	£6,123	£5,462
75%	£11,865	£11,095	£10,325	£9,554	£8,784	£8,013	£7,243	£6,473	£5,702
80%	£12,986	£12,106	£11,225	£10,345	£9,464	£8,584	£7,703	£6,823	£5,942
85%	£14,106	£13,116	£12,125	£11,135	£10,144	£9,154	£8,163	£7,173	£6,182
90%	£15,227	£14,126	£13,026	£11,925	£10,825	£9,724	£8,624	£7,523	£6,422
95%	£16,568	£15,467	£14,366	£13,266	£12,165	£11,065	£9,964	£8,864	£7,763
100%	£17,908	£16,808	£15,707	£14,607	£13,506	£12,405	£11,305	£10,204	£9,104

Where the net benefit produces a non-negative outcome then it is cost effective for the NHS to adopt the indicator.

When this is the case, the cells are highlighted with a yellow background.

Appendix E: Net Benefit Base Case Analysis

Pilot 4 - Referral to Pulmonary Rehab for COPD: Net Benefit Analysis

Value per point achieved	£133.76	Societal value of a QALY	£20,000		
Number of practices	8,228				
Mean practice population	6,297				
		**Basline achievement**			
Minimum threshold	40%	Eligible population (mean % of practice population)	0.750%		£462
Maximum threshold	90%	Baseline achievement (mean % of eligible patients)	25.0%		0.024

National totals

Expected Achievement	QOF payments (£000s)									Change in treatment cost (£)	Change in QALYs
**Points**	2	3	4	5	6	7	8	9	10		
30%	£0	£0	£0	£0	£0	£0	£0	£0	£0	£8,976,380	466
35%	£0	£0	£0	£0	£0	£0	£0	£0	£0	£17,952,760	933
40%	£0	£0	£0	£0	£0	£0	£0	£0	£0	£26,929,139	1399
45%	£220	£330	£440	£550	£660	£770	£880	£991	£1,101	£35,905,519	1865
50%	£440	£660	£880	£1,101	£1,321	£1,541	£1,761	£1,981	£2,201	£44,881,899	2332
55%	£660	£991	£1,321	£1,651	£1,981	£2,311	£2,641	£2,972	£3,302	£53,858,279	2798
60%	£880	£1,321	£1,761	£2,201	£2,641	£3,082	£3,522	£3,962	£4,402	£62,834,659	3264
65%	£1,101	£1,651	£2,201	£2,751	£3,302	£3,852	£4,402	£4,953	£5,503	£71,811,038	3730
70%	£1,321	£1,981	£2,641	£3,302	£3,962	£4,622	£5,283	£5,943	£6,603	£80,787,418	4197
75%	£1,541	£2,311	£3,082	£3,852	£4,622	£5,393	£6,163	£6,934	£7,704	£89,763,798	4663
80%	£1,761	£2,641	£3,522	£4,402	£5,283	£6,163	£7,044	£7,924	£8,805	£98,740,178	5129
85%	£1,981	£2,972	£3,962	£4,953	£5,943	£6,934	£7,924	£8,915	£9,905	£107,716,558	5596
90%	£2,201	£3,302	£4,402	£5,503	£6,603	£7,704	£8,805	£9,905	£11,006	£116,692,937	6062
95%	£2,201	£3,302	£4,402	£5,503	£6,603	£7,704	£8,805	£9,905	£11,006	£125,669,317	6528
100%	£2,201	£3,302	£4,402	£5,503	£6,603	£7,704	£8,805	£9,905	£11,006	£134,645,697	6995

Net Benefit (£000s)

	2	3	4	5	6	7	8	9	10
30%	£350	£350	£350	£350	£350	£350	£350	£350	£350
35%	£699	£699	£699	£699	£699	£699	£699	£699	£699
40%	£1,049	£1,049	£1,049	£1,049	£1,049	£1,049	£1,049	£1,049	£1,049
45%	£1,179	£1,069	£959	£849	£739	£629	£518	£408	£298
50%	£1,308	£1,088	£868	£648	£428	£208	-£12	-£232	-£453
55%	£1,438	£1,108	£778	£448	£117	-£213	-£543	-£873	-£1,203
60%	£1,568	£1,127	£687	£247	-£193	-£634	-£1,074	-£1,514	-£1,954
65%	£1,697	£1,147	£597	£46	-£504	-£1,054	-£1,604	-£2,155	-£2,705
70%	£1,827	£1,167	£506	-£154	-£815	-£1,475	-£2,135	-£2,796	-£3,456
75%	£1,956	£1,186	£416	-£355	-£1,125	-£1,896	-£2,666	-£3,436	-£4,207
80%	£2,086	£1,206	£325	-£555	-£1,436	-£2,316	-£3,197	-£4,077	-£4,958
85%	£2,216	£1,225	£235	-£756	-£1,746	-£2,737	-£3,727	-£4,718	-£5,708
90%	£2,345	£1,245	£144	-£956	-£2,057	-£3,158	-£4,258	-£5,359	-£6,459
95%	£2,695	£1,594	£494	-£607	-£1,707	-£2,808	-£3,908	-£5,009	-£6,110
100%	£3,045	£1,944	£844	-£257	-£1,358	-£2,458	-£3,559	-£4,659	-£5,760

Where the net benefit produces a non-negative outcome then it is cost effective for the NHS to adopt the indicator.

When this is the case, the cells are highlighted with a yellow background.

Appendix F: Net Benefit Analysis Assuming No Reduction in Secondary Healthcare Costs

Pilot 4 - Referral to Pulmonary Rehab for COPD: Net Benefit Analysis

Value per point achieved	£133.76	Societal value of a QALY	£20,000
Number of practices	8,228		
Mean practice population	6,297		

Basline achievement

Minimum threshold	40%	Eligible population (mean % of practice population)	0.750%	£954
Maximum threshold	90%	Baseline achievement (mean % of eligible patients)	25.0%	0.024

**Points**	2	3	4	5	6	7	8	9	10

National totals

Expected Achievement	QOF payments (£000s)									Change in treatment cost (£)	Change in QALYs
30%	£0	£0	£0	£0	£0	£0	£0	£0	£0	£18,535,641	466
35%	£0	£0	£0	£0	£0	£0	£0	£0	£0	£37,071,283	933
40%	£0	£0	£0	£0	£0	£0	£0	£0	£0	£55,606,924	1399
45%	£220	£330	£440	£550	£660	£770	£880	£991	£1,101	£74,142,566	1865
50%	£440	£660	£880	£1,101	£1,321	£1,541	£1,761	£1,981	£2,201	£92,678,207	2332
55%	£660	£991	£1,321	£1,651	£1,981	£2,311	£2,641	£2,972	£3,302	£111,213,848	2798
60%	£880	£1,321	£1,761	£2,201	£2,641	£3,082	£3,522	£3,962	£4,402	£129,749,490	3264
65%	£1,101	£1,651	£2,201	£2,751	£3,302	£3,852	£4,402	£4,953	£5,503	£148,285,131	3730
70%	£1,321	£1,981	£2,641	£3,302	£3,962	£4,622	£5,283	£5,943	£6,603	£166,820,773	4197
75%	£1,541	£2,311	£3,082	£3,852	£4,622	£5,393	£6,163	£6,934	£7,704	£185,356,414	4663
80%	£1,761	£2,641	£3,522	£4,402	£5,283	£6,163	£7,044	£7,924	£8,805	£203,892,055	5129
85%	£1,981	£2,972	£3,962	£4,953	£5,943	£6,934	£7,924	£8,915	£9,905	£222,427,697	5596
90%	£2,201	£3,302	£4,402	£5,503	£6,603	£7,704	£8,805	£9,905	£11,006	£240,963,338	6062
95%	£2,201	£3,302	£4,402	£5,503	£6,603	£7,704	£8,805	£9,905	£11,006	£259,498,980	6528
100%	£2,201	£3,302	£4,402	£5,503	£6,603	£7,704	£8,805	£9,905	£11,006	£278,034,621	6995

Net Benefit (£000s)

30%	-£9,210	-£9,210	-£9,210	-£9,210	-£9,210	-£9,210	-£9,210	-£9,210	-£9,210
35%	-£18,419	-£18,419	-£18,419	-£18,419	-£18,419	-£18,419	-£18,419	-£18,419	-£18,419
40%	-£27,629	-£27,629	-£27,629	-£27,629	-£27,629	-£27,629	-£27,629	-£27,629	-£27,629
45%	-£37,058	-£37,168	-£37,278	-£37,388	-£37,498	-£37,609	-£37,719	-£37,829	-£37,939
50%	-£46,488	-£46,708	-£46,928	-£47,148	-£47,368	-£47,588	-£47,809	-£48,029	-£48,249
55%	-£55,918	-£56,248	-£56,578	-£56,908	-£57,238	-£57,568	-£57,899	-£58,229	-£58,559
60%	-£65,347	-£65,787	-£66,228	-£66,668	-£67,108	-£67,548	-£67,989	-£68,429	-£68,869
65%	-£74,777	-£75,327	-£75,877	-£76,428	-£76,978	-£77,528	-£78,079	-£78,629	-£79,179
70%	-£84,206	-£84,867	-£85,527	-£86,188	-£86,848	-£87,508	-£88,169	-£88,829	-£89,489
75%	-£93,636	-£94,407	-£95,177	-£95,947	-£96,718	-£97,488	-£98,259	-£99,029	-£99,799
80%	-£103,066	-£103,946	-£104,827	-£105,707	-£106,588	-£107,468	-£108,349	-£109,229	-£110,109
85%	-£112,495	-£113,486	-£114,476	-£115,467	-£116,458	-£117,448	-£118,439	-£119,429	-£120,420
90%	-£121,925	-£123,026	-£124,126	-£125,227	-£126,327	-£127,428	-£128,529	-£129,629	-£130,730
95%	-£131,135	-£132,235	-£133,336	-£134,436	-£135,537	-£136,637	-£137,738	-£138,839	-£139,939
100%	-£140,344	-£141,445	-£142,545	-£143,646	-£144,746	-£145,847	-£146,948	-£148,048	-£149,149

Where the net benefit produces a non-negative outcome then it is cost effective for the NHS to adopt the indicator.

When this is the case, the cells are highlighted with a yellow background.

Appendix G: Net Benefit Analysis Assuming 50% Reduction in Utility

Pilot 4 - Referral to Pulmonary Rehab for COPD : Net Benefit Analysis

Value per point achieved	£130.51	Societal value of a QALY	£20,000
Number of practices	8,228		
Mean practice population	6,297		

Basline achievement

Minimum threshold	40%	Eligible population (mean % of practice population)	0.750%	£454
Maximum threshold	90%	Baseline achievement (mean % of eligible patients)	25.0%	0.012

National totals

Expected Achievement	QOF payments (£000s)									Change in treatment cost (£)	Change in QALYs
**Points**	2	3	4	5	6	7	8	9	10		
30%	£0	£0	£0	£0	£0	£0	£0	£0	£0	£8,820,945	233
35%	£0	£0	£0	£0	£0	£0	£0	£0	£0	£17,641,889	466
40%	£0	£0	£0	£0	£0	£0	£0	£0	£0	£26,462,834	699
45%	£215	£322	£430	£537	£644	£752	£859	£966	£1,074	£35,283,779	933
50%	£430	£644	£859	£1,074	£1,289	£1,503	£1,718	£1,933	£2,148	£44,104,723	1166
55%	£644	£966	£1,289	£1,611	£1,933	£2,255	£2,577	£2,899	£3,222	£52,925,668	1399
60%	£859	£1,289	£1,718	£2,148	£2,577	£3,007	£3,436	£3,866	£4,295	£61,746,613	1632
65%	£1,074	£1,611	£2,148	£2,685	£3,222	£3,758	£4,295	£4,832	£5,369	£70,567,557	1865
70%	£1,289	£1,933	£2,577	£3,222	£3,866	£4,510	£5,154	£5,799	£6,443	£79,388,502	2098
75%	£1,503	£2,255	£3,007	£3,758	£4,510	£5,262	£6,013	£6,765	£7,517	£88,209,446	2332
80%	£1,718	£2,577	£3,436	£4,295	£5,154	£6,013	£6,873	£7,732	£8,591	£97,030,391	2565
85%	£1,933	£2,899	£3,866	£4,832	£5,799	£6,765	£7,732	£8,698	£9,665	£105,851,336	2798
90%	£2,148	£3,222	£4,295	£5,369	£6,443	£7,517	£8,591	£9,665	£10,738	£114,672,280	3031
95%	£2,148	£3,222	£4,295	£5,369	£6,443	£7,517	£8,591	£9,665	£10,738	£123,493,225	3264
100%	£2,148	£3,222	£4,295	£5,369	£6,443	£7,517	£8,591	£9,665	£10,738	£132,314,170	3497

Net Benefit (£000s)

30%	-£4,158	-£4,158	-£4,158	-£4,158	-£4,158	-£4,158	-£4,158	-£4,158	-£4,158
35%	-£8,316	-£8,316	-£8,316	-£8,316	-£8,316	-£8,316	-£8,316	-£8,316	-£8,316
40%	-£12,474	-£12,474	-£12,474	-£12,474	-£12,474	-£12,474	-£12,474	-£12,474	-£12,474
45%	-£16,846	-£16,954	-£17,061	-£17,168	-£17,276	-£17,383	-£17,491	-£17,598	-£17,705
50%	-£21,219	-£21,434	-£21,649	-£21,863	-£22,078	-£22,293	-£22,508	-£22,722	-£22,937
55%	-£25,592	-£25,914	-£26,236	-£26,558	-£26,880	-£27,202	-£27,525	-£27,847	-£28,169
60%	-£29,964	-£30,394	-£30,823	-£31,253	-£31,682	-£32,112	-£32,542	-£32,971	-£33,401
65%	-£34,337	-£34,874	-£35,411	-£35,948	-£36,485	-£37,022	-£37,558	-£38,095	-£38,632
70%	-£38,710	-£39,354	-£39,998	-£40,643	-£41,287	-£41,931	-£42,575	-£43,220	-£43,864
75%	-£43,082	-£43,834	-£44,586	-£45,337	-£46,089	-£46,841	-£47,592	-£48,344	-£49,096
80%	-£47,455	-£48,314	-£49,173	-£50,032	-£50,891	-£51,750	-£52,609	-£53,468	-£54,327
85%	-£51,828	-£52,794	-£53,760	-£54,727	-£55,693	-£56,660	-£57,626	-£58,593	-£59,559
90%	-£56,200	-£57,274	-£58,348	-£59,422	-£60,496	-£61,569	-£62,643	-£63,717	-£64,791
95%	-£60,358	-£61,432	-£62,506	-£63,580	-£64,653	-£65,727	-£66,801	-£67,875	-£68,949
100%	-£64,516	-£65,590	-£66,664	-£67,738	-£68,811	-£69,885	-£70,959	-£72,033	-£73,107

Where the net benefit produces a non-negative outcome then it is cost effective for the NHS to adopt the indicator.

When this is the case, the cells are highlighted with a yellow background.

Appendix H: Net Benefit Analysis Assuming Lower Estimate for Eligible Population

Pilot 4 - Referral to Pulmonary Rehab for COPD: Net Benefit Analysis

Value per point achieved	£133.76	Societal value of a QALY	£20,000
Number of practices	8,228		
Mean practice population	6,297		

Basline achievement

Minimum threshold	40%	Eligible population (mean % of practice population)	0.375%	£462
Maximum threshold	90%	Baseline achievement (mean % of eligible patients)	25.0%	0.024

National totals

Expected Achievement	QOF payments (£000s)									Change in treatment cost (£)	Change in QALYs
**Points**	2	3	4	5	6	7	8	9	10		
30%	£0	£0	£0	£0	£0	£0	£0	£0	£0	£4,488,190	233
35%	£0	£0	£0	£0	£0	£0	£0	£0	£0	£8,976,380	466
40%	£0	£0	£0	£0	£0	£0	£0	£0	£0	£13,464,570	699
45%	£220	£330	£440	£550	£660	£770	£880	£991	£1,101	£17,952,760	933
50%	£440	£660	£880	£1,101	£1,321	£1,541	£1,761	£1,981	£2,201	£22,440,949	1166
55%	£660	£991	£1,321	£1,651	£1,981	£2,311	£2,641	£2,972	£3,302	£26,929,139	1399
60%	£880	£1,321	£1,761	£2,201	£2,641	£3,082	£3,522	£3,962	£4,402	£31,417,329	1632
65%	£1,101	£1,651	£2,201	£2,751	£3,302	£3,852	£4,402	£4,953	£5,503	£35,905,519	1865
70%	£1,321	£1,981	£2,641	£3,302	£3,962	£4,622	£5,283	£5,943	£6,603	£40,393,709	2098
75%	£1,541	£2,311	£3,082	£3,852	£4,622	£5,393	£6,163	£6,934	£7,704	£44,881,899	2332
80%	£1,761	£2,641	£3,522	£4,402	£5,283	£6,163	£7,044	£7,924	£8,805	£49,370,089	2565
85%	£1,981	£2,972	£3,962	£4,953	£5,943	£6,934	£7,924	£8,915	£9,905	£53,858,279	2798
90%	£2,201	£3,302	£4,402	£5,503	£6,603	£7,704	£8,805	£9,905	£11,006	£58,346,469	3031
95%	£2,201	£3,302	£4,402	£5,503	£6,603	£7,704	£8,805	£9,905	£11,006	£62,834,659	3264
100%	£2,201	£3,302	£4,402	£5,503	£6,603	£7,704	£8,805	£9,905	£11,006	£67,322,848	3497

Net Benefit (£000s)

30%	£175	£175	£175	£175	£175	£175	£175	£175	£175
35%	£350	£350	£350	£350	£350	£350	£350	£350	£350
40%	£525	£525	£525	£525	£525	£525	£525	£525	£525
45%	£479	£369	£259	£149	£39	-£71	-£181	-£291	-£401
50%	£434	£214	-£6	-£226	-£446	-£666	-£887	-£1,107	-£1,327
55%	£389	£59	-£272	-£602	-£932	-£1,262	-£1,592	-£1,922	-£2,253
60%	£344	-£97	-£537	-£977	-£1,417	-£1,858	-£2,298	-£2,738	-£3,178
65%	£298	-£252	-£802	-£1,353	-£1,903	-£2,453	-£3,003	-£3,554	-£4,104
70%	£253	-£407	-£1,068	-£1,728	-£2,388	-£3,049	-£3,709	-£4,369	-£5,030
75%	£208	-£563	-£1,333	-£2,103	-£2,874	-£3,644	-£4,415	-£5,185	-£5,955
80%	£163	-£718	-£1,598	-£2,479	-£3,359	-£4,240	-£5,120	-£6,001	-£6,881
85%	£117	-£873	-£1,864	-£2,854	-£3,845	-£4,835	-£5,826	-£6,816	-£7,807
90%	£72	-£1,028	-£2,129	-£3,230	-£4,330	-£5,431	-£6,531	-£7,632	-£8,733
95%	£247	-£854	-£1,954	-£3,055	-£4,155	-£5,256	-£6,357	-£7,457	-£8,558
100%	£422	-£679	-£1,779	-£2,880	-£3,980	-£5,081	-£6,182	-£7,282	-£8,383

Where the net benefit produces a non-negative outcome then it is cost effective for the NHS to adopt the indicator.

When this is the case, the cells are highlighted with a yellow background.
